# Self-care for anxiety and depression: a comparison of evidence from Cochrane reviews and practice to inform decision-making and priority-setting

**DOI:** 10.1186/s12906-020-03038-8

**Published:** 2020-08-10

**Authors:** Karen Pilkington, Lisa Susan Wieland

**Affiliations:** 1 School of Health and Care Professions, University of Portsmouth, James Watson West, 2 King Richard 1st Road, Portsmouth, P01 2FR UK; 2 Center for Integrative Medicine, University of Maryland School of Medicine, 520 West Lombard Street, East Hall, Baltimore, MD 21201 USA

**Keywords:** Anxiety disorders, Depression, Depressive disorder, Evidence-based practice, Self-care, Systematic reviews

## Abstract

**Background:**

Self-care refers to a range of activities and approaches undertaken by an individual to maintain health and manage ill-health which may include various complementary or alternative approaches. The purpose of this study was to identify the self-care approaches used by the general public for depression and anxiety, assess the usefulness of Cochrane reviews for informing decisions on self-care and highlight any gaps in the evidence.

**Methods:**

Searches were carried out for surveys of self-care for anxiety and/or depression and for Cochrane reviews and protocols of interventions with potential for use in self-care. Data was extracted from each review and Plain Language Summaries assessed for content, consistency and readability. Interventions reported in surveys and in Cochrane reviews were compared and effectiveness of each assessed.

**Results:**

Surveys from 10 countries reported a variety of self-care interventions, 17 of which appeared in 2 or more surveys and which included dietary supplements, herbal medicines, mind-body therapies and various forms of exercise. Twenty-two reviews and 5 protocols on potential self-care interventions were identified, the majority in depression. Twelve interventions were judged effective or promising, most with small effect sizes. Readability of summaries was highly variable: half were written at college/university level. Several commonly used approaches were not covered by Cochrane reviews.

**Conclusions:**

This study has revealed the interventions currently used by the general public which are judged effective or promising based on Cochrane reviews. Some disparity is highlighted between interventions used in practice and the availability of reliable evidence, and in the presentation of effectiveness and safety. Being able to direct patients to reliable, accessible information is a positive step in ensuring effective patient-centered, evidence-informed care. Addressing gaps, ensuring consistency and increasing usability of evidence intended for the general public will support this goal.

## Background

The World Health Organization (WHO) defines self-care as “the ability of individuals, families and communities to promote health, prevent disease, maintain health, and to cope with illness and disability with or without the support of a health-care provider” [1]. The WHO has proposed a global perspective on self-care in traditional medicine which encompasses a range of approaches and activities. These include health promotion and illness prevention, healthy lifestyle including diet and physical activities, techniques that may promote health such as acupressure and massage, exercises for health including qigong, tai chi and yoga, and meditation for mental and spiritual wellbeing, in addition to herbal and other traditional remedies for disease management [1]. Based on this definition, it is clear that there is potential for a range of complementary approaches to contribute to self-care.

There is, however, some variation in how the term self-care is understood and applied in practice with several terms used to describe the role that individuals may take in assessing and managing their own health. ‘Self-management’ is considered to relate particularly to long-term health conditions and is often implemented as an ongoing partnership between the patient and his/her health-care providers [2]. In contrast, some organisations use the term ‘self-care’ to refer solely to the efforts a healthy individual takes to maintain health and prevent illness [2] while others perceive self-diagnosis/testing to fall within the scope of self-care [3]. A review of self-care in mental health suggests that it is a ‘broad, inclusive concept, not distinct from but encompassing those related concepts of recovery, self-management and selfhelp’ [4]. Nevertheless, according to the WHO definition, appropriate self-medication and self-treatment are key components of self-care [1].

Many countries are placing increasing emphasis on the decision-making and actions taken by individuals to manage their own health. In the UK, health policy has continued to focus on self-management/self-care in the context of promoting personal responsibility for health [5]. In Japan, self-help is seen as a potential, albeit partial, solution to the financial burden of an aging population which is placing increasing demands on central funding of healthcare [6]. Self-care is also being increasingly adopted in China with decision-making influenced by self-perceived illness status, economic circumstances, and education [7]. Web-based research has demonstrated significant yet comparable levels of self-care (40–55% of respondents) in Australia, Japan, UK and US suggesting that the trend is not influenced by the health care provision model [8].

Self-care takes on particular importance in the field of mental health due to issues related to access to treatment. Individuals with mental health conditions are often reluctant to seek medical advice [9–11] despite considerable efforts to address the perceived stigma around conditions such as depression and anxiety [12]. Those experiencing these problems may be unwilling to take or adhere to pharmacological treatments [13] or unable to access relevant psychological therapies [14, 15]. Indeed, lack of treatment for anxiety disorders and depression has been shown to be a global issue [14].

For those seeking to manage their own anxiety or depression, whether through choice or circumstance, a whole array of treatments or activities are on offer. Within the field of mental health, some authors have distinguished between informal approaches: ‘simple things an individual can do on their own without the need for professional guidance’ and guided self-help involving professional supervision [16]. Those recovering from depression may have to ‘navigate’ a range of pharmacological and psychological therapies as well as support groups [17], and health services are not perceived to offer help in guiding self-treatment and selecting approaches from the huge number of options that are available [18]. Whether self-care is facilitated and overseen by a health professional, or is self-motivated and self-monitored, there is a requirement for reliable information on effectiveness and safety. It is particularly important for individuals to have access to comprehensive and understandable information when the strategies are self-chosen in order to make informed decisions and, before using a self-care health intervention, to ensure that it does no harm to the individual [19, 20].

Cochrane reviews are widely recognized as the most reliable form of evidence on specific health interventions. Topics covered by Cochrane reviews are largely those proposed by the review teams working in that particular area. The target audience for Cochrane reviews is broad: as the website states ‘Cochrane is for anyone who is interested in using high-quality information to make health decisions. Whether you are a doctor or nurse, patient or carer, researcher or funder … ’, (https://www.cochrane.org/about-us) Thus, it is conceivable, and indeed intended, that patients (and /or members of the general public) may seek to base their decision-making around the conclusions of Cochrane reviews. To support this, each review has a ‘plain language summary’ on the first page. However, it is not clear to what extent Cochrane reviews address potential self-care approaches and whether the format would support and inform self-care by those with conditions such as anxiety and depression.

The aim of this study was to identify the approaches used most commonly in self-care of anxiety and depression through collating results of surveys of use, to assess evidence from Cochrane reviews on potential self-care interventions for depression and anxiety and the usefulness of the reviews for informing self-care of depression and anxiety, and to ascertain potential ‘gaps’ in Cochrane evidence for commonly used or recommended self-care approaches to treat or prevent these conditions.

## Materials and methods

A novel approach was taken that was developed specifically for this study and which has the potential to be applied in other areas and contexts. The most commonly used or recommended self-care strategies for depression or anxiety are likely to vary in different countries and health care systems. As Cochrane is a global organisation providing evidence across national boundaries, we aimed to identify the most commonly used or recommended strategies across countries. To achieve this, we produced a ‘snapshot’ by collating results from published international, national or regional surveys on the topic. We then identified Cochrane reviews on potential self-care interventions for depression or anxiety. We assessed the review characteristics and conclusions, and how the conclusions were presented to the public, before comparing the topic coverage between the surveys and the Cochrane reviews and presenting the findings of the Cochrane reviews.

### Searching and selection of surveys describing self-care for depression and/or anxiety

To identify self-care approaches commonly used or recommended for depression or anxiety, we searched PubMed, MEDLINE, EMBASE, AMED and PsycINFO for surveys reporting use of self-care interventions using the search strategies reported in Appendix 1. Initial searches for surveys were carried out in April 2017; these were updated in February 2019 and updated and extended in April 2020. Surveys were selected that reported use or endorsement of specific self-care approaches on a regional, national or international basis specifically for anxiety and/or depression. Surveys were excluded that: only reported use by category e.g. non-conventional/complementary versus conventional approaches; were conducted within a single organization (e.g. one clinic or health centre) only; where use was in anxious/depressed populations but not specifically for anxiety or depression. Conference abstracts and dissertations were also excluded. Each set of search results was screened by one author (KP) to select possibly relevant surveys. Full-texts of these were obtained and any surveys where relevance was unclear were screened by both authors with any disagreements on inclusion resolved by discussion.

### Selection of self-care interventions for depression and/or anxiety

**Fig. 1:**
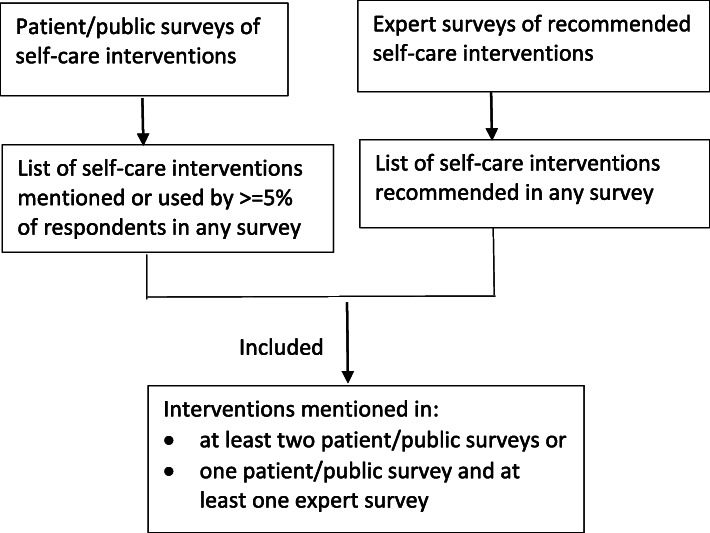
Framework for identification of commonly used or recommended self-care interventions

To generate a list of self-care interventions known to be used frequently in practice, we extracted all self-care approaches to the treatment or prevention of depression and anxiety that were mentioned in any survey, focusing on those that were endorsed by at least 5% of survey respondents. We also sought evidence of expert recommendations on effective self-help therapies for depression or anxiety. We considered self-care approaches that were endorsed in two or more surveys of consumers or in one consumer study plus one expert study, to be those most commonly used or recommended for depression or anxiety. We compared the list of these approaches to the interventions covered in relevant Cochrane reviews. The selection process is summarised in Fig. 1.

### Searching and selection of Cochrane reviews on self-care for depression and/or anxiety

We screened all published reviews from the Cochrane Common Mental Disorders Review Group (CCMD) or grouped under the topic of ‘depression’ or ‘anxiety’ on the Cochrane Library webpage (cochranelibrary.com/cdsr/reviews/topics) to identify reviews on potential self-care interventions for preventing or treating depression or anxiety. Initial searching was conducted in April 2017, and repeated in March 2018, February 2019, and March 2020 for new or updated records. We defined self-care interventions as interventions that could be selected and applied without the assistance of a practitioner or professional. We included interventions that could involve practitioner guidance, but did not necessarily do so (e.g., vitamins, yoga), and included only self-care interventions for risk factors, symptoms or medical diagnoses of depression or anxiety in adults (age 18 and older). We excluded the following reviews: those of interventions for trauma-related conditions (e.g., post-traumatic stress disorder), obsessive-compulsive disorder, panic disorder, bipolar disorder, or any other conditions not specifically described as depression or anxiety; those of interventions that relied upon either conventional or alternative practitioners (e.g., prescription medicine, talk therapy, acupuncture); reviews focused on children and young people, even if some trials may have included people aged over 18 years; reviews that had been withdrawn from publication. Protocols that clearly covered self-care were included as an indication of research in progress and limited data was extracted from these. Protocols were excluded if it was unclear whether the final review would include self-care interventions.

Both authors independently screened the title and abstract of each review, and each review selected by one or both authors as possibly relevant was then independently screened in full by both authors. Disagreements surrounding inclusion were resolved by discussion.

### Information extracted from Cochrane reviews on self-care for depression and/or anxiety

We extracted descriptive data from included reviews into an Excel spreadsheet. Information included the country of the corresponding author, sources of external funding, date of the latest review update, the condition (depression or anxiety), the approach (treatment or prevention), the population, the type of self-care intervention, the main comparisons, whether any meta-analyses were done, and the review conclusions. We independently categorized the interventions as effective (where the review concluded there was evidence of a beneficial effect), promising (where there was some evidence but this was limited e.g. one or two trials, or low quality, and not conclusive) or unclear (where the evidence did not provide a clear indication of effectiveness or otherwise) based on the conclusions. We then compared decisions and agreed a final category for each intervention. We also assessed the readability of the plain language summary of each review with the Flesch Reading Ease test, which generates a readability score based upon the word and sentence length [21]. Higher scores on the Flesch Reading Ease test indicate text that is easier to read. Scores of 60 or above indicate plain English that is readable by the average adult, scores of 30 to 60 indicate text that is readable by advanced or college students, and scores below 30 indicate that text is readable by college/university graduates [22].

### Comparison of data from surveys with Cochrane reviews

We compared the list of interventions compiled from collation of the surveys with the interventions assessed within the Cochrane reviews and presented this comparison in tabular form.

## Results

### Surveys on self-care for depression and/or anxiety

**Fig. 2:**
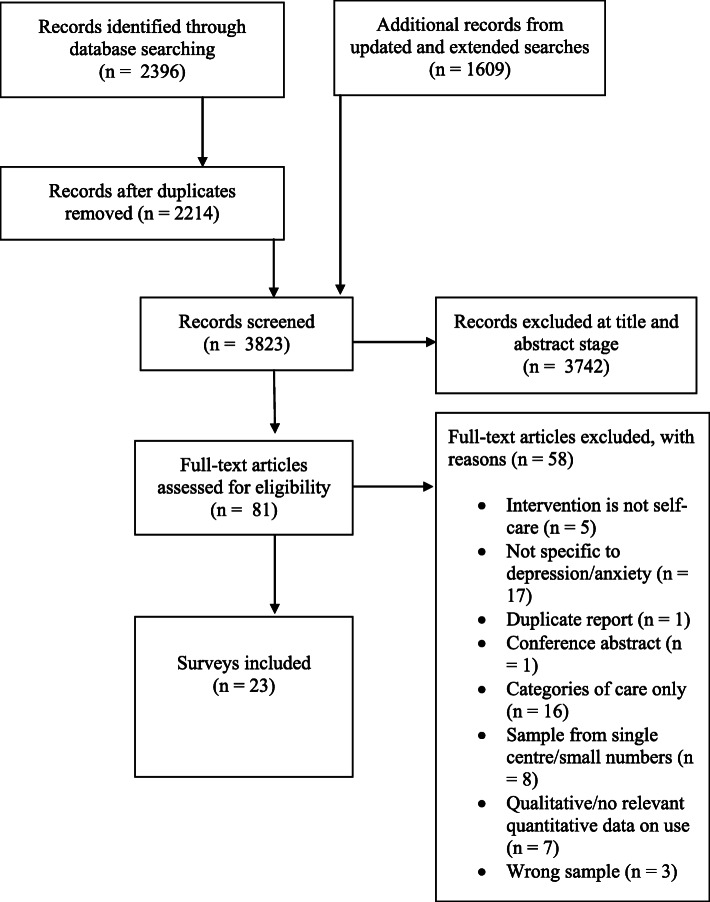
PRISMA-style flowchart describing screening of surveys

The initial searches for surveys of self-care retrieved a total of 2396 records of which 182 were duplicates; additional and extended searches in April 2020 retrieved an additional 1609 records so that the total screened was 3823. The screening process is summarized in Fig. 2. Twenty surveys of patient or public recommendations or use of self-care interventions for depression and/or anxiety were included, 5 from Australia [23–27], 1 from Austria [28], 2 from Germany [29, 30], 2 from Italy [31, 32], 1 from Korea [33], 1 from the Netherlands [34], 1 from Portugal [35], 2 from Taiwan [36, 37], and 5 from the USA [38–42]. The surveys were online, postal, telephone, or face-to-face surveys of consumers or the general public, in which opinions were solicited on self-help strategies to manage symptoms of depression (*n* = 15), or both depression and anxiety (*n* = 5). We also found three surveys involving recommendations for self-help strategies from experts [43–45].

One expert survey [44] from Australia used a Delphi method to solicit recommendations for effective self-care strategies for sub-threshold depression from international panels of depression consumers and clinicians. An additional survey by the same research team used similar methods to identify recommendations for effective self-care strategies for anxiety [43]. We extracted a list of 10 interventions from the strategies that were listed in these studies as endorsed by at least 80% of both consumers and clinicians (a cut-off point used by the authors when assessing consensus): bibliotherapy, dietary change, exercise, internet self-help, meditation, mindfulness, pets/animals, relaxation, self-help groups, and exposure to sunlight. We also found a third survey carried out among Norwegian psychologists, in which bibliotherapy and internet self-help were both commonly recommended for patients with depression or anxiety [45]. See Table 1 for a summary of both the patient or public surveys and the expert surveys included for this overview.

**Table 1:** Surveys collecting information on use and recommendations for self-care interventions in depression or anxiety

Author year	Country	Sample size	Sample and methods
**Surveys describing self-care interventions used for depression or anxiety (*n*** **= 20)**			
Jorm 2004 [23]	Australia	*N* = 6618	Postal survey of community sample of adults in Canberra and south-east New South Wales, collecting information on actions taken to cope with depression during the past 6 months.
Olesen 2010 [27]	Australia	*N* = 8841	Nationally representative face-to-face survey of Australian adults, collecting information on self-management strategies for a diagnosed affective or anxiety disorder during the past 12 months.
Parker 2007 [24]	Australia	*N* = 2692	Online survey of Australian adults who has experienced depression, investigating perceived effectiveness of self-help and other strategies.
Parslow 2004 [26]	Australia	*N* = 7485	Postal survey of community sample of adults in the Canberra environs, collecting information on use of CAM to treat symptoms of depression or anxiety.
Proudfoot 2015 [25]	Australia	*N* = 465	National online survey of Australian men investigating positive strategies to prevent and manage depression
Holzinger 2012 [28]	Austria	*N* = 1205	Telephone survey of Viennese adults assessing help-seeking and treatment recommendations in response to a vignette depicting a case of moderate depression.
Lowe 2006 [29]	Germany	*N* = 87	Face-to-face interview of outpatients with depression, investigating attitudes and preferences for self-management to improve mental well-being.
Riedel-Heller 2005 [30]	Germany	*N* = 2516	Nationally representative face-to-face interview of adults collecting information on preferred treatment options in response to a vignette representing major depressive disorder
Carta 2014 [31]	Italy	*N* = 1200	Telephone survey of Sardinian adults assessing help-seeking and treatment recommendations in response to a vignette depicting a case of depression.
Munizza 2013 [32]	Italy	*N* = 1001	Telephone survey of Italian adults assessing beliefs and attitudes regarding depression etiology and treatment.
Shin 2014 [33]	Korea	*N* = 1214	Online survey of national sample of adults from the community, patients with sub-threshold or mild depression, and psychiatrists about the use and helpfulness of self-help for depression.
Loureiro 2013 [35]	Portugal	*N* = 4938	Supervised written survey of Portuguese young people presented with a vignette depicting depression and asked questions concerning self-help strategies.
Hsu 2009 [36]	Taiwan	*N* = 201	Telephone survey of Taiwanese adults recently discharged from psychiatric hospitalization, collecting information on CAM use for depression.
Tsai 2006 [37]	Taiwan	*N* = 220	Face-to-face interviews of elderly nursing home residents, investigating self-care strategies to manage depressive symptoms.
Van Grieken 2018 [34, 46]	The Netherlands	*N* = 193	Online survey assessing use and perceived helpfulness of self-management strategies in participants recently recovered from an episode of major depression.
Bazargan 2008 [39]	USA	*N* = 315	Face-to-face interview regarding frequency and type of CAM use for depression among sample of primarily African American and Hispanic individuals at clinics in Los Angeles, California screening positive for mild to severe depression.
Bystritsky 2012 [40]	USA	*N* = 1004	Telephone survey of CAM therapies to help with ‘mood or energy’ among primary care patients diagnosed with an anxiety disorder and participating in a randomized trial (CALM).
Grzywacz 2002 [42]	USA	*N* = 5827	In-person survey of a national sample of older adults (age 65+) collecting information on CAM use to treat mental health.
Kessler 2001 [38]	USA	*N* = 266	Telephone survey of nationally representative sample of adults on CAM therapies used for treatment of self-defined ‘anxiety attacks’ or ‘severe depression’ during the previous 12 months.
Musil 2017 [41]	USA	*N* = 335	Mailed survey assessing self-management of depression symptoms among Ohio grandmothers with self-identified depression.
**Surveys recommending self-care interventions for depression or anxiety (*n*** **= 3)**			
Morgan 2009 [44]	Australia	*N* = 97	Delphi survey of international panel of experts and consumers on recommended interventions for sub-threshold depression
Morgan 2016 [43]	Australia	*N* = 83	Delphi survey of international panel of experts and consumers on recommended interventions for sub-threshold anxiety
Nordgreen 2011 [45]	Norway	*N* = 815	Online survey of Norwegian psychologists, collecting information on self-help strategies recommended to patients with anxiety or depression.

We selected 17 strategies mentioned by at least 5% of respondents in at least two of the 20 patient or public surveys: aromatherapy, bibliotherapy, dietary improvement, exercise, fish oil/omega-3, herbal medicine (most frequently St. John’s wort (Hypericum perforatum)), homeopathy, internet self-help, massage, meditation/mindfulness, music, pets, prayer/spirituality, relaxation, self-help groups, vitamins/minerals, and yoga. We selected an additional two strategies recommended in one of the three expert surveys and by at least 5% of respondents in one of the 20 patient or public surveys: natural environments and sunlight exposure. We therefore identified a total of 19 strategies that we considered to be commonly used or recommended. See Table 2 for a listing of each of the self-help therapies from patient/public surveys or expert surveys.

**Table 2:** Self-care interventions reported in surveys

Intervention	Patient/public use for depression or anxiety or both?	Number of surveys mentioning intervention (***N*** = 20)	Recommendations from expert surveys
Aromatherapy	Depression	2	–
Bibliotherapy	Depression	4	Depression/anxiety
Dietary change†	Depression	6	Depression/anxiety
Dietary supplements (other than vitamins/minerals or herbs) ‡	Depression	5	–
Exercise	Depression	11	Depression/anxiety
Herbal medicine§	Depression/anxiety	10	Depression
Homeopathy	Depression	3	–
Internet self-help	Depression	3	Depression
Massage	Depression/anxiety	7	–
Meditation/mindfulness	Depression	7	Depression/anxiety
Music	Depression	3	–
Natural environments	Depression	1	Anxiety
Pets/animals	Depression	3	Depression
Prayer/spirituality	Depression	4	–
Relaxation	Depression/anxiety	10	Depression/anxiety
Self-help groups (not internet)	Depression	5	Depression
Sunlight exposure	Depression	1	Depression
Vitamins/minerals¶	Depression/anxiety	7	–
Yoga	Depression	6	Anxiety

†Changes included reducing caffeine, increasing caffeine, eating a high-carbohydrate diet, and reducing sugar, consuming cocoa or chocolate, improved diet

‡Non-vitamin, non-mineral, non-herbal dietary supplements and specific supplements including fish oil/omega-3, L-tryptophan or 5HTP, and SAMe

§Herbal medicine in general and specific herbs including St. John’s wort and chamomile

¶ Vitamins in general, magnesium and vitamin-B complex

### Cochrane reviews on self-care for depression and/or anxiety

**Fig. 3:**
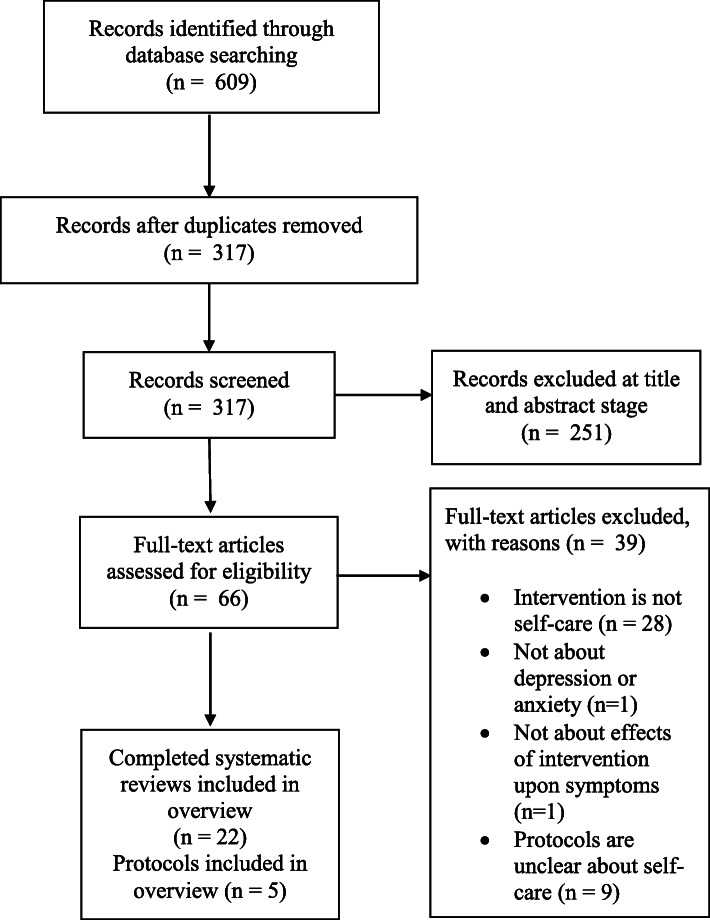
PRISMA-style flowchart describing screening of systematic reviews

We identified 62 records from the Cochrane Library browse by topic list of reviews on Mental Health/Anxiety Disorders and 116 records from the Cochrane Library browse by topic list of reviews on Mental Health/Depression. We identified 228 records from the Cochrane Library browse by Review Group list of CCMD reviews. Finally, we checked the internal Cochrane database (Archie) for further review documents belonging to CCMD and identified 320 review documents with CD numbers indicating publication in the Cochrane Library. These CD numbers retrieved 239 records from the Cochrane Library. After repeating searches in 2018, 2019 and 2020, we retrieved a total of 609 records of which 292 were duplicates. We screened a total of 317 records and included a total of 22 reviews [47–67] and five protocols [68–72] (see Fig. 3).

The included protocols and reviews were published between 2004 and 2019. Twenty focused on depression including seasonal affective disorder [47, 49–51, 54, 55, 57, 58, 60–62, 64–72], 6 focused on anxiety [48, 52, 53, 59, 63, 73], 1 covered both depression and anxiety [56]. Four of the reviews and three of the protocols focused on prevention of depression or anxiety, one of the reviews focused on both prevention and treatment of depression, and the remaining 17 reviews and two protocols focused on treatment of depression or anxiety. Interventions were primarily herbal or dietary supplements. Twelve therapies were judged effective or promising, most with small effect sizes. Those judged effective were exercise, relaxation and St John’s wort for depression. Tryptophan and 5-hydroxytryptophan for depression and kava extract for anxiety were judged effective but associated with serious adverse effects precluding use. ‘Promising’ interventions included bibliotherapy, internet-based self-help, meditation/mindfulness for anxiety and specific dietary/herbal supplements (folate, omega-3, passiflora, S-adenosyl methionine) for depression. Insufficient evidence was available on light therapy, inositol, valerian and selenium. Cochrane reviews of the following have not been completed: aromatherapy, dietary change, homeopathy, massage, music, natural environments, pets/animals, prayer/spirituality, self-help groups (not internet) or yoga. See Table 3 for a description of review characteristics.

**Table 3:** Characteristics of included systematic reviews

**Title (completed reviews)**	Year*	**Self-care intervention**
Comparative effectiveness of continuation and maintenance treatments for persistent depressive disorder in adults [64]	2019	Herbal (St. John’s wort)
Light therapy for preventing seasonal affective disorder [66]	2019	Light therapy
Melatonin and agomelatine for preventing seasonal affective disorder [67]	2019	Dietary supplement (melatonin)
Psychosocial interventions for preventing and treating depression in dialysis patients [65]	2019	Acupressure, Exercise, Mind-body (meditation, relaxation, spiritual practice), Social activity
Interventions for treating anxiety after stroke [63]	2017	Mind-body (relaxation)
S-adenosyl methionine (SAMe) for depression in adults [62]	2016	Dietary supplement (SAMe)
Omega-3 fatty acids for depression in adults [61]	2015	Dietary supplement (Omega-3 fatty acids)
Media-delivered cognitive behavioural therapy and behavioural therapy (self-help) for anxiety disorders in adults [59]	2013	Psychological individual self-help
Dietary supplements for preventing postnatal depression [60]	2013	Dietary supplements (selenium yeast, EPA, DHA)
Exercise for depression [58]	2013†	Exercise
Psychosocial interventions for prevention of psychological disorders in law enforcement officers [56]	2008	Mind-body (relaxation), Exercise
Psychotherapeutic treatments for older depressed people [57]	2008	Psychological individual self-help (bibliotherapy)
Relaxation for depression [54]	2008	Mind-body (relaxation)
St John’s wort for major depression [55]	2008^†^	Herbal (St John’s wort)
Passiflora for anxiety disorder [73]	2007	Herbal (Passiflora)
Valerian for anxiety disorders [53]	2006	Herbal (Valerian)
Meditation therapy for anxiety disorders [52]	2006	Mind-body (meditation)
Light therapy for non-seasonal depression [51]	2004	Light therapy
Inositol for depressive disorders [50]	2004	Dietary supplement (Inositol)
Folate for depressive disorders [49]	2003	Dietary supplement (Folate)
Kava extract for treating anxiety [48]	2003	Herbal (Kava)
Tryptophan and 5-Hydroxytryptophan for depression [47]	2002	Dietary supplement (Tryptophan and 5-Hydroxytryptophan)
**Title (protocols)**	**Year**	**Self-care intervention**
Antidepressants for major depression disorder in older people: a network meta-analysis [72]	2019	Dietary supplement (Tryptophan)
Interventions (other than psychosocial, psychological and pharmacological) for treating postpartum depression [71]	2019	Mind-body (e.g. bright light therapy, physical exercise, yoga, sleep deprivation); Dietary supplements (e.g. omega-3 fatty acids); Herbal (e.g. St. John’s Wort).
The process and delivery of cognitive behavioural therapy (CBT) for depression in adults: a network meta-analysis [70]	2018	Multimedia CBT including self-help, Self-help CBT without multimedia
Multimedia-delivered cognitive behavioural therapy versus face-to-face cognitive behavioural therapy for depression in adults [69]	2018	Multimedia CBT including self-help
Prevention of depression in chronically physically ill adults [68]	2014	Psychological individual self-help (bibliotherapy)

*Year of most recent version of published review

†Last search for trials in 2013 and review has been split into two reviews for updating

The readability of summaries varied considerably. The Flesch Reading Ease score for the plain language summaries of completed reviews ranged from 7.3 to 41.9 (median 29.75). Four summaries had Flesch Reading Ease scores lower than 20, and seven summaries had scores between 20 and 30, indicating that half of review plain language summaries were written on a readability level most suitable for college/university graduates.

Information in the PLS on risks/safety of interventions was limited (see Table 4). Ten reviews made no mention in the summaries of adverse events or potential harms (two of these reviews found no relevant studies) [50, 54, 56–60, 63, 64, 67]. Six review summaries stated explicitly that available information on harms was missing or limited from the included studies, without presenting any information on numbers or types of events that was available from the studies [52, 53, 61, 65, 66, 73]. Six review summaries discussed adverse events specifically [47–49, 51, 55, 62], and, of these, one review mentioned that the intervention was effective but potentially dangerous [47] while three summaries made a comparison between harms from the self-care intervention and some other active intervention, in one case based on one trial [53] and in the others on several trials [55, 62].

**Table 4:** Comparison of interventions from surveys with relevant Cochrane reviews

Self-Care interventions mentioned in multiple patient/public or expert surveys	Focus of relevant Cochrane review(s)	Assessment of effectiveness based on Plain Language Summary (PLS) †	Safety information in the PLS
Aromatherapy	–		
Bibliotherapy	media-delivered cognitive or behavioural therapy for anxiety disorders	+	–
	psychotherapeutic treatments for older depressed people	No PLS	–
Dietary change	–		
Dietary supplements (other than vitamins/minerals or herbs)	tryptophan and 5-Hydroxytryptophan for depression	++	Mentions side effects that have occurred; also that tryptophan associated with development of a fatal condition
	S-adenosyl methionine for depression	+	Mention of fewer side effects than with an antidepressant
	omega-3 fatty acids for depression	+	Mentions that insufficient high quality evidence to determine … negative side effects
	dietary supplements (EPA or DHA) for preventing postpartum depression	?	–
	melatonin for preventing seasonal affective disorder	No evidence	–
Exercise	exercise for depression	++	–
	psychosocial interventions to prevent psychological disorders in law enforcement personnel	No mention of exercise in PLS	–
	psychosocial interventions to prevent and treat depression in dialysis patients	++	Mentions adverse events very uncertain.
Herbal medicine	kava extract for treating anxiety	++	Mentions that few adverse events were reported in the reviewed trials *
	passiflora for anxiety disorders	+	Mentions that not possible to draw any conclusions on the safety
	St John’s wort for major depression	++	Mention of fewer side effects than antidepressants; that side effects are usually minor and uncommon but effects of other drugs might be significantly compromised.
	valerian for anxiety disorders	?	Mentions that tolerated as well as an anxiolytic but that further studies are needed for conclusions on safety.
	continuation and maintenance treatments for persistent depressive disorder in adults	No mention of St. John’s wort in PLS	–
Homeopathy	–		
Internet-based self-help	media-delivered cognitive or behavioural therapy for anxiety disorders	+	–
Massage	–		
Meditation/mindfulness	meditation therapy for anxiety disorders	+	Mentions that adverse effects have not been reported
	psychosocial interventions to prevent and treat depression in dialysis patients	?	Mentions adverse events very uncertain.
Music	–		
Natural environments	–		
Pets/animals	–		
Prayer/spirituality	psychosocial interventions to prevent and treat depression in dialysis patients	?	Mentions adverse events very uncertain.
Relaxation	relaxation for depression	++	–
	interventions for treating anxiety after stroke	?	–
	psychosocial interventions to prevent psychological disorders in law enforcement personnel	No mention of relaxation in PLS	–
	psychosocial interventions to prevent and treat depression in dialysis patients	++	Mentions adverse events very uncertain.
Self-help groups (not internet)	–		
Sunlight exposure	light therapy** for preventing seasonal affective disorder	?	Mentions that the included study provided no information on side effects.
	light therapy** for non-seasonal depression	?	Mentions a potential adverse effect that needs to be considered.
Vitamins/minerals	folate for depressive disorders	+	Mentions that it was well-tolerated in the included trials.
	inositol for depressive disorders	?	–
	dietary supplements (selenium) for preventing postpartum depression	?	–
Yoga	–		

†Key for statements in review PLS: -- no review; ++ effective; + promising;? unclear

* Comments on this review include report of possible liver toxicity

** Light therapy uses machines that simulate components of natural outdoor light

### Comparison between strategies described in surveys and in Cochrane reviews

A comparison between the surveys and Cochrane reviews reveals that several dietary supplements addressed by Cochrane reviews (omega-3, S-adenosyl methionine (SAMe), tryptophan) appeared in only 1 survey; some (inositol and melatonin) were not specifically mentioned in any survey. Conversely, many types of self-care treatments mentioned in multiple surveys (aromatherapy, music, yoga) were not addressed in any Cochrane reviews. Some survey interventions were covered by Cochrane reviews but the review question was limited to specific populations (e.g., bibliotherapy for elderly persons, relaxation to treat anxiety after stroke, exercise for dialysis patients) or covered only one indication when surveys indicated likely use for another indication (e.g., there is a Cochrane review of meditation for anxiety but surveys report that consumers might also use meditation for depression). Several reviews were excluded from this overview because they focused on interventions that could be used in self-care according to surveys, but the Cochrane review specifically focused on provision by a practitioner (e.g., music therapy for depression). Table 4 summarizes the interventions and corresponding Cochrane reviews.

## Discussion

Self-care is increasingly emphasized by healthcare organizations as an approach to manage the ever-extending healthcare burden. It is also sought by many, particularly those with mental health conditions such as anxiety and depression. Informed decision-making does, however, require access to reliable information. Cochrane reviews provide coverage of self-care approaches and are a potentially useful source on these for health professionals and the general public. This study, using a novel and pragmatic approach, has highlighted where Cochrane reviews could be used to inform decision-making by patients or guidance by health professionals on frequently used self-care approaches. It has also highlighted the fact that there is currently some disparity between what is used in practice and the availability of reliable evidence. If the recommendations of organizations outside conventional healthcare are also taken into account, then the disparity may be greater [74]. Thus, there are clear evidence gaps to be filled by conducting and/or updating Cochrane reviews on those approaches used in practice. This will provide a more comprehensive resource for health care professionals aiming to guide patients through the possible options available. For resources such as Cochrane reviews to be optimal in informing self-care, there is, however, a need to ensure that the Plain Language Summary of each review is written using a consistent approach to content and organization and an appropriate reading level to allow maximum accessibility to patients and consumers. Achieving an optimal balance between transparency through detailed reporting of systematic reviews and an accessible, user-friendly summary is a considerable challenge. Nevertheless, if lay summaries suggest that intervention is safe and possibly effective, then it is important that the review should also contain accessible information on the intervention that allows the consumer to understand whether the review is applicable to his/her context. The results of this study suggest that providing the information in a way that allows comparison of more than one intervention would also be valuable. Currently, comparison between different therapies and approaches for a particular condition is neither easy nor quick.

As self-care may incorporate elements of self-diagnosis and self-monitoring, clearly written information on potential risks of treatments is also needed, with careful consideration on how this is worded and how might it be applied by members of the general public. For the practicing clinician, there is a dilemma to be addressed in that some of the more frequently used self-care approaches do not fit with current guidance but directly discouraging their use may also discourage their disclosure in consultations. Thus, being able to direct patients to reliable, accessible information on non-conventional as well as more conventional treatments or to use this a basis for discussions may be a positive step in ensuring effective patient-centered, evidence-informed care.

Limitations of this study include the fact that relying upon published surveys is an untested method for identifying the most commonly used or recommended interventions. The surveys were also heterogeneous in terms of sample, design and overall aims. Relying on the surveys we found and then using a practical rule of thumb (percentage of respondents reporting a therapy; at least two surveys mentioning a therapy) almost certainly resulted in omission of some strategies that are frequently used. However, our finding that there were no Cochrane reviews on many of the therapies that we did identify is not affected by this limitation. Additionally, identifying all relevant surveys proved challenging. It is possible that relevant surveys were not identified even though search strategies were specifically designed for this purpose and were based on preliminary scoping and testing of search terms. Reporting of the results of surveys was highly variable and some potentially relevant surveys had to be excluded as it was not possible to extract data on specific self-help therapies. Another limitation of this research is that we did not directly test the readability of the Cochrane plain language summaries but rather relied upon a commonly used rubric of readability, the Flesch Reading Ease test. Future exploration of this issue should directly test how comprehensible Cochrane plain language summaries are to readers and to what extent they answer their most pressing questions. Finally, the Cochrane reviews were selected as the ‘gold standard’ evidence on treatments but the quality of individual Cochrane reviews may vary. Furthermore, when Cochrane evidence on an intervention does not exist or is outdated, it is still possible that other reviews may have high-quality and up-to-date evidence on the topic. We focused on Cochrane not only because Cochrane reviews are generally considered to be methodologically rigorous and unbiased, but also because Cochrane aims to be a ‘one-stop shop’ for evidence and to translate this evidence into formats suitable for consumers as well as clinicians or researchers. Other well-conducted systematic reviews exist that are not part of the Cochrane Library and these do provide summaries of the evidence but these are generally less accessible to patients and consumers, and are not part of a globally available and updated database.

## Conclusion

This study has revealed the interventions currently used in practice by the general public which were judged effective or promising based on Cochrane reviews. These include exercise, and relaxation for depression; bibliotherapy, internet based self-help and meditation for anxiety). Several herbs and nutritional supplements also fall into these categories. It has also highlighted the fact that there is currently some disparity between self-care approaches used in practice and the availability of reliable evidence. Greater clarity and consistency in presenting conclusions on safety is required, particularly for herbal and nutritional supplements. Being able to direct patients to reliable, accessible information on non-conventional as well as more conventional treatments is a positive step in ensuring effective patient-centered, evidence-informed care. The results of this study suggest that efforts should be made to address gaps, align evidence reviews with practice and ensure the consistency of evidence intended for the general public. The novel approach used in this study could be applied more widely to aid priority setting for systematic review topics, particularly as the emphasis increases on supporting evidence-based self-care.

## Data Availability

The data that support the findings of this study are available from the survey and systematic review publications cited in the paper. The data extraction from these sources is available from the corresponding author upon reasonable request.

## Appendix

### Search strategies for identifying surveys of self-care for anxiety or depression

PubMed:

((depression [Title] OR depressive [Title]) OR (anxiety [Title] or anxious [Title]) AND (usage [Title] OR survey [Title/Abstract]) AND (self-care [Title] OR self-treat*[Title] OR self-manage*[Title] OR self-prescri*[Title] OR complementary [Title] OR alternative [Title] OR over-the-counter [Title] OR OTC [Title]))

OR

((self-manage*[Title/Abstract] OR self-treat*[Title/Abstract] OR self-care [Title/Abstract] OR self-prescri*[Title/Abstract] OR over-the-counter [Title/Abstract] OR OTC [Title/Abstract] OR complementary therapies [MeSH Terms]) AND (“Anxiety”[MeSH] OR “Anxiety Disorders”[MeSH] OR “Depression”[MeSH] OR “Depressive Disorder”[MeSH]) AND (“Surveys and Questionnaires”[MeSH] OR “Health Care Surveys”[MeSH] OR “Health Surveys”[MeSH]))

AMED (Ovid):

(depression OR depressive OR anxiety OR anxious) AND (usage OR survey) AND (self-care OR self-treat* OR self-manage* OR self-prescri* OR complementary OR alternative OR over-the-counter OR OTC) in title *or abstract*.*

EMBASE (Ovid): As per AMED.

PsycInfo (EBSCO):

(depression OR depressive OR anxiety OR anxious) AND (usage (title) OR survey (title/abstract)) AND (self-care OR self-treat* OR self-manage* OR self-prescri* OR complementary OR alternative OR over-the-counter OR OTC) in title or abstract.

* Term in italics added to most recent search to extend strategy.

## Abbreviations

AMED: Allied and Complementary Medicine Database
CAM: Complementary and alternative medicine
CCMD: Cochrane Common Mental Disorders Review Group
CD: Cochrane Database
CBT: Cognitive behavioural therapy
DHA: Docosahexaenoic acid
EPA: Eicosapentaenoic acid
5HTP: 5-Hydroxytryptophan
NHS: National Health Service
PLS: Plain Language Summary
PRISMA: Preferred Reporting Items for Systematic Reviews and Meta-Analyses
SAMe: S-adenosyl methionine
UK: United Kingdom
US: United States
